# Co-dispersal of the blood fluke *Schistosoma japonicum* and *Homo sapiens* in the Neolithic Age

**DOI:** 10.1038/srep18058

**Published:** 2015-12-21

**Authors:** Mingbo Yin, Hong-Xiang Zheng, Jing Su, Zheng Feng, Donald P. McManus, Xiao-Nong Zhou, Li Jin, Wei Hu

**Affiliations:** 1 State Key Laboratory of Genetic Engineering and Ministry of Education Key Laboratory of Contemporary Anthropology, Collaborative Innovation Center of Genetics and Development, School of Life Sciences, Fudan University, Shanghai, 200438, China; 2 National Institute of Parasitic Diseases, Chinese Center for Disease Control and Prevention, Key Laboratory of Parasite and Vector Biology of the Chinese Ministry of Health, WHO Collaborating Center for Malaria, Schistosomiasis and Filariasis, Shanghai, 200025, China; 3 QIMR Berghofer Medical Research Institute, Brisbane, Queensland 4006, Australia; 4 Chinese Academy of Sciences Key Laboratory of Computational Biology, CAS-MPG Partner Institute for Computational Biology, SIBS, CAS, Shanghai, 200021, China

## Abstract

The global spread of human infectious diseases is of considerable public health and biomedical interest. Little is known about the relationship between the distribution of ancient parasites and that of their human hosts. *Schistosoma japonicum* is one of the three major species of schistosome blood flukes causing the disease of schistosomiasis in humans. The parasite is prevalent in East and Southeast Asia, including the People’s Republic of China, the Philippines and Indonesia. We studied the co-expansion of *S. japonicum* and its human definitive host. Phylogenetic reconstruction based on complete mitochondrial genome sequences showed that *S. japonicum* radiated from the middle and lower reaches of the Yangtze River to the mountainous areas of China, Japan and Southeast Asia. In addition, the parasite experienced two population expansions during the Neolithic agriculture era, coinciding with human migration and population growth. The data indicate that the advent of rice planting likely played a key role in the spread of schistosomiasis in Asia. Moreover, the presence of different subspecies of *Oncomelania hupensis* intermediate host snails in different localities in Asia allowed *S. japonicum* to survive in new rice-planting areas, and concurrently drove the intraspecies divergence of the parasite.

The global spread of human infectious diseases is of considerable biomedical interest as this process relies on the dispersal of both host and pathogen. Indeed, the current Ebola outbreaks caused by viruses of the genera *Ebolavirus* and *Marburgvirus* resulted from the spread of their human hosts^1^. Previous studies have also reported geographically structured populations for a number of human pathogens^2^,^3^,^4^,^5^,^6^, some of which have been linked to ancient human host migrations^3^,^4^,^5^,^6^. However, for many parasites, which may have several hosts in their life cycle, there is no information as to whether they migrated with human or other hosts.

Schistosomes are ancient parasites with two distinct hosts during their lifecycle: an intermediate fresh water snail host and a definitive human or other mammalian host. These blood flukes cause schistosomiasis which ranks as the second most serious human parasitic infection globally in terms of burden of disease estimates. There are three major species that infect humans: *Schistosoma mansoni, S. haematobium and S. japonicum.* The latter schistosome has exploited a wide range of at least 46 species of mammalian definitive hosts, including humans and a variety of wild and domesticated animals, but uses only *Oncomelania hupensis* as its intermediate host^7^. *S. japonicum* is prevalent in the People’s Republic of China, particularly in the marsh and lake regions along the Yangtze River basin (Hunan, Hubei, Jiangxi, Anhui and Jiangsu province) and in mountainous areas (Sichuan and Yunnan province), and in parts of the Philippines and Indonesia^8^,^9^. The population genetic structure of *S. japonicum* distributed in East Asia still remains unclear. Several studies of genetic variation, based on the use of mitochondrial (mt) genes and microsatellite loci as gene markers, have shown that in mainland China worms from the lake regions and from the mountain regions are different^10^. However, there is limited information regarding the relationship between Chinese mainland *S. japonicum* and other strains of the parasite in East Asia^11^. Where did the ancestor of all strains of the *S. japonicum* group originate? How and when did the different strains diverge during the course of evolutionary history? Why did *S. japonicum* expand into new areas and hosts? Addressing these questions will provide an understanding of the evolution of this parasite and shed light on its history of disease transmission.

We collected 119 *S. japonicum* samples from 13 locations endemic for Asiatic schistosomiasis, including Japan, Indonesia, Philippines, Taiwan and mainland China, and we investigated the co-expansion of *S. japonicum* and its human definitive host. Phylogenetic reconstruction based on complete mitochondrial genome sequences showed that *S. japonicum* radiated from the middle and lower reaches of the Yangtze River to the mountainous areas of China, Japan and Southeast Asia. In addition, the parasite experienced two population expansions during the Neolithic agricultural era, coinciding with human migration and population expansion. The data indicate that the advent of rice planting likely played a key role in the spread of schistosomiasis japonica in Asia.

## Results

### Haplotype analysis

**Figure 1:**
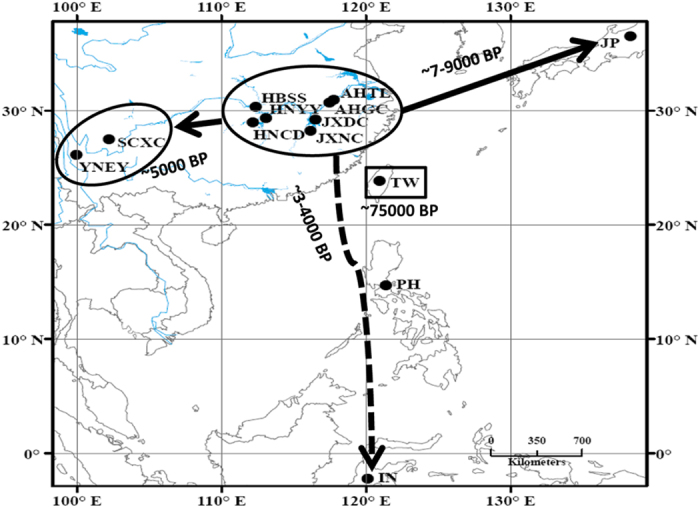
Location of the *Schistosoma japonicum* samples used in analysis. The arrows indicate the direction of spread of *S. japonicum*. For abbreviations of each sample, refer to Table S1. The migration times are annotated in the figure (BP: years before present). The Map was created using ArcGIS® version 10.2 software by Esri http://www.esri.com/software/arcgis).

**Figure 2:**
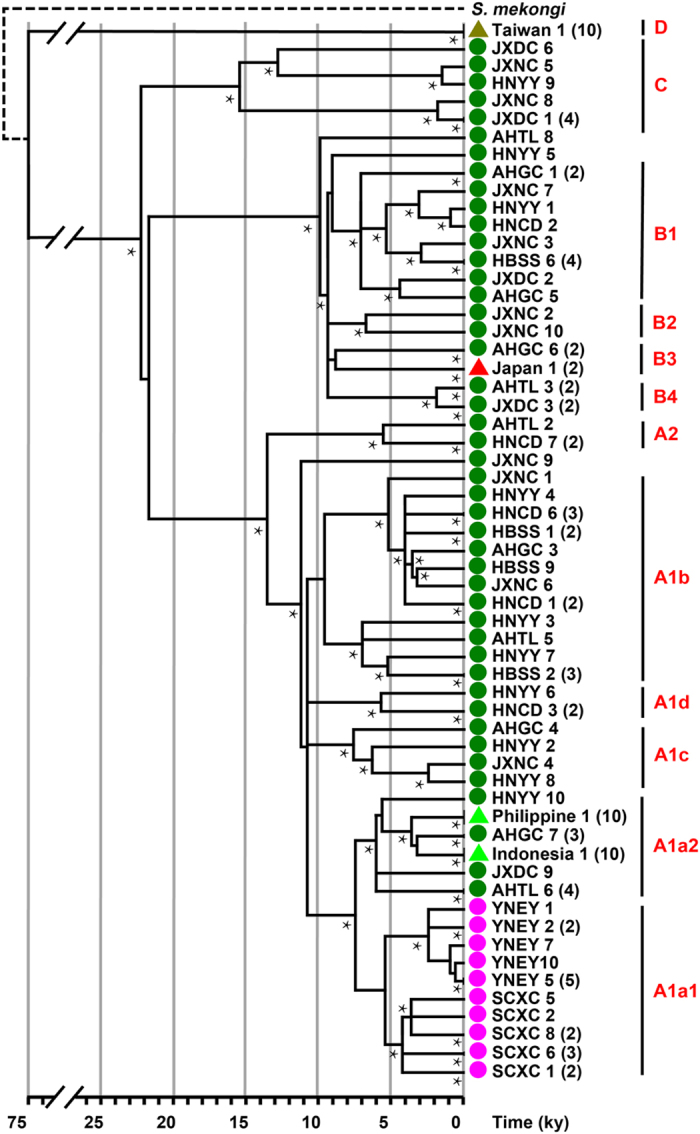
Phylogenetic tree of 119 complete *Schistosoma japonicum* mt genomes, based on maximum likelihood and Bayesian methods. The *S. japonicum* phylogeny was calibrated with the mt genome of *S. mekongi,* a close relative found exclusively in the Mekong river basin of Laos and Cambodia in South-east Asia. Nodes with high statistical support (>80% approximate likelihood branch support in ML analysis and >0.9 posterior probability in Bayesian analysis, respectively) are highlighted by asterisks. The numbers in brackets indicate the number of identical mt genome sequences obtained. The green circles indicate lake samples, the pink circles indicate samples collected from mountainous areas of mainland China, whereas the coloured triangles represent samples from Chinese Taiwan, Japan, the Philippines and Indonesia. For the abbreviations of each sample, refer to Table S1.

We collected *S. japonicum* samples from Indonesia, Japan, the Philippines, Chinese Taiwan and nine locations on mainland China, including two populations from mountainous areas and seven from the lake regions (for detailed information, see Fig. 1 and Table S1). In total, 119 complete *S. japonicum* mitochondrial DNA (mtDNA) genomes (~14-kilobase) were sequenced successfully using Next-Generation Sequencing (NGS) technology, with high quality (the coverage ranged from 571 to 6,593, with the average being 3,588; Fig. S1). For *S. japonicum* lineages from Indonesia, Japan, the Philippines and Taiwan, only one haplotype was detected per population based on scrutiny of their complete mtDNA genomes (Fig. 2), indicating an extremely low effective population size (N_e_) in these groups. However, lower nucleotide diversity was evident in the two *S. japonicum* populations from the mountainous areas of China compared with those from the lake areas (Table S1 and Fig. 1). In total, 45 haplotypes were distributed among *S. japonicum* populations from the lake areas of mainland China indicating a very rich level of genetic variation.

### Phylogeny reconstruction

The phylogenetic history of the 119 complete mtDNA sequences obtained for the *S. japonicum* samples was inferred by a Bayesian model. The topology of the phylogeny was further confirmed by both the maximum likelihood (ML) method and median-joining network (refer to Table S2 and Fig. S2 for details of the mtDNA sequence variations). Important nodes of the topology showed relative high support (posterior probability by the Bayesian method and approximate likelihood support by the maximum likelihood method). In total, 23 *S. japonicum* haplogroups were determined and their defining variants (Fig. S3) were classified into four major haplogroups (i.e. Haplogroup A, B, C and D) (Fig. 2). First of all, *S. japonicum* samples from Taiwan (Haplogroup D) diverged out from mainland lineages. In addition, the distance between the Taiwan and the ancestor of non-Taiwan lineages (Haplogroup A'B'C) showed ~6 times the average genetic distance non-Taiwan lineages diverging from their most recent common ancestor. Large divergence indicated a considerable difference in schistosome infection, which coincided with the fact that non-Taiwan *S. japonicum* can infect humans while Taiwan lineage do not.

*S. japonicum* from mainland of China was mainly composed of 3 major haplogroups which almost diverged tridently. Haplogroup C split out from Haplogroup A′B′C little earlier than Haplogroup A and B, which separated after additional 3 variants (Fig. S3). Japan and Southeast Asian samples belonged to Haplogroup A and B, respectively. Mountain region lineages showed a definite monophyletic clade, belonging to Haplogroup A. According to the phylogeny, we detected 878 variant sites, 1055 substitutions and 30 indels (Table S2). High nucleotide diversity (π) in the *ND5* gene was revealed by the analysis of sliding window of 200 bp (step size = 50 bp) along the entire genome (Fig. S2). A similar plot of distribution on total substitutions showed an unexpected high mutation hot spot at the end of the mitochondrial genome. After inspection, we found that substitutions occurred relatively frequently at 3 sites in the whole phylogeny (13971, 7 times, 13980, 8 times, 13981, 9 times, respectively). Interestingly, variants of these sites were linked 7 times but the mutation mechanisms involved require further research.

### Inference of reference sequence

According to the phylogenetic tree (Fig. 2), the lineages of Taiwan *S. japonicum*, which does not colonize humans^12^, diverged first from the other lineages that can infect humans (Fig. 2). The extant *S. japonicum* reference mt genome is 14085 bp (Genbank access: NC_002544). Compared with the extant reference mtDNA genome sequence, we detected a haplotype bearing 43 nucleotide mismatches with two additional insertions (a guanine ‘insertion’ at position 2318 and a thymine ‘insertion’ at position 2450) which all the human-colonizing lineages originated from. The guanine insertion at 2318 encodes COX3, which affects the codon frame and results in an extended 2 amino acids compared with the original protein product. The N-terminal of the corrected COX3 protein subunit was more similar to that of *S. mekongi*. The thymine insertion at 2450 encodes tRNA^Glu^. Both insertions were also found in other published *S. japonicum* mtDNA sequences^13^. In addition, the original *S. japonicum* mtDNA reference showed many mismatches in nucleotides compared to the extant sequences. Considering the potential error of the extant reference sequence and the phylogenetic significance, we proposed to use the reconstructed sequence, which was the ancestor of all human-infecting lineages (ancestor of Haplogroup A, B and C, i.e., the defining sequence of Haplogroup A'B'C) and to renumber the reference to 14087 bp (Supporting information). Mismatches to the new reference sequence showed the derived allele in *S. japonicum* evolution, which would have a practical influence.

### Estimation of divergence

For the calibration point, we assumed that the divergence of *S. japonicum* from the related *S. mekongi*, found in Laos and Cambodia, occurred 3.8 million years ago (mya) according to a recent study^14^. Then, we employed two strategies, a ML method via PAML package v4.7 and a Bayesian method via BEAST v1.8, to estimate the ages of the *S. japonicum* haplogroups (Fig. S4 and Table S3) and the mutation rates (Table S4). In both analyses, whole genome sequences partitioned in six regions were used. For comparison, protein coding sequences were employed in ML analysis considering that gene rearrageenment might cause alignment problem between *S. japonicum* and *S. mekongi*. In addition, mtDNA was shaped by selective constraints, which affected the time estimates based on whole genome sequences. However, synonymous mutation was considered free of the pressure. In the phylogeny of *S. japonicum*, there were 529 variants on the third codon, of which only 27 variants were non-synonymous mutations. Thus, we also used the third position of the protein codon to estimate an approximately ‘neutral’ rate in ML analysis.

Generally, in total, four age estimates via ML and Bayesian strategies were obtained for each haplogroup and these did not show large discrepancies (Fig. S4 and Table S3). Haplogroup D exhibited a large genetic distance compared with the others and diverged into Taiwanese *S. japonicum* isolates ~75 thousand years ago (kya), whereas Haplogroups A, B and C separated much more recently and almost simultaneously, about 22 kya. Moreover, Haplogroup C split out a little earlier than Haplogroup A or B, and the Haplogroups A'B and A'B'C were only differentiated in several hundred years. Two star-like lineages (Haplogroup B and A1) took place ~10 kya, showing a great expansion in *S. japonicum*. In Haplogroup B, Japan lineages split from mainland China ~7–9 kya. In Haplogroup A1a, *S. japonicum* migrated to Southeast Asia ~3–4 kya and to mountain regions ~5 kya. Two star-like lineages (A1b1 and A1a1a) were within ~5 kya and indicated recent expansions in the agriculture era. Mutation rate estimates showed that the non-coding region evolved fastest for free of selective constraints while rRNA and tRNA were relatively conservative (Table S4).

### Inference of population expansions

**Figure 3:**
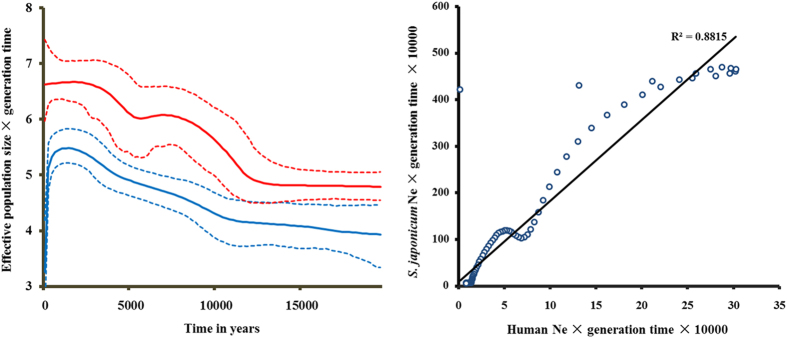
Demographic analysis of *Schistosoma japonicum* and *Homo sapiens*. (**A**) Bayesian skyline plots of *Schistosoma japonicum* (blue) and three lineages of *Homo sapiens* (red). The x-axis is the time from present in units of years, and the y-axis is the product of maternal effective size and generation time. The solid line is the median estimate and the dashed lines show the 95% highest posterior density limits. (**B**) Correlations of effective population size between *Homo sapiens* and *S. japonicum*. The linear regression line and coefficient were denoted.

Given the information we obtained on the phylogeny and haplogroup age of *S. japonicum*, we further reconstructed its demographic history using BSP via mtDNA coding regions (Fig. 3 and Table S5). This showed that the effective population size (N_e_) of *S. japonicum* expanded from 15,000 to 50,000 at ~11 to 7 kya, assuming half a year as the length of generation time. This expansion corresponded with two star-like lineages (Haplogroup B and A1; Fig. 2) both coalescing at ~10 kya. Interestingly, *S. japonicum* showed an additional growth peak ~5–2 kya in the agricultural era, with N_e_ ranging from 70,000 to 300,000, which might correlate with an expansion in the lineages A1b1, A1a2, A1a1b and A1a1a (Fig. S3). For comparison, we also employed three human mtDNA lineages (B5, M7 and F) to construct human BSP for comparison. Haplogroup B5, M7 and F were major haplogroups in the Southern part of East Asia and then some sub-lineages migrated to Southeast Asia, probably corresponding to the human expansion due to rice agriculture in Southern China (Fig. 3 and Table S5). Human BSP also showed two peaks of rapid Ne growth. The former was a ten-fold increase ~12–8 kya, followed by a subsequent three-fold expansion ~5–3 kya in the agriculture era. Human BSP correlated well with *S. japonicum* BSP (Fig. 3), indicating that *S. japonicum* expanded accompanying human activity, especially the advent of the agricultural era. The latter expansion of *S. japonicum* appeared much greater than the former, and corresponded to more human migration events.

## Discussion

In this work, we constructed the phylogeny tree based on whole mitochondrial genome sequences and four major haplogroups (i.e. Haplogroup A, B, C and D; Fig. 2) were determined for *S. japonicum*. All the Taiwanese lineages were placed in Haplogroup D, the Japanese lineages were placed in Haplogroup B and the lineages from Southeast Asia were represented in Haplogroup A. In addition, the lineages from the Chinese lake regions had the highest diversity, spreading out into all the lineages of Haplogroup C and some sub-haplogroups of A and B. The lineages from the mountainous regions of China were clearly clustered, presenting as a specific sub-haplogroup of A, which we termed Haplogroup A1a1 (Figs 1 and 2). The reason that the lake worms showed high diversity are gene flow happened in this area because of the rich waternet, while the lineages from the mountainous regions of China and the island strain showed low diversity as result of their geographic isolation. It was very clear that the Taiwan samples generated an independent clade which was separated from the other human colonized worms. This result may coincide with its unique infection feature. Therefore, the 14,087 bp mtDNA reference genome (see Supporting information) of *S. japonicum* we constructed represents the ancestor of all human-colonizing lineages. This new reference sequence will have practical and phylogenetic importance in future research as the potential mismatches could help in the determination of derived alleles in *S. japonicum* evolution.

Furthermore, the ages of the *S. japonicum* haplogroups were estimated; the Haplogroup D exhibited a large genetic distance compared with the others and diverged into Taiwanese *S. japonicum* isolates ~75 thousand years ago (kya), whereas Haplogroups A, B and C separated much more recently and almost simultaneously, about 22 kya. Moreover, Haplogroup C split out a little earlier than Haplogroup A or B, and the Sub-haplogroups A'B and A'B'C were only differentiated in several hundred years. In addition, the Japanese lineages in Haplogroup B split from the Chinese *S. japonicum* mainland lake area lineages ~7–9 kya, and *S. japonicum* from the lake regions in Haplogroup A1a migrated to Southeast Asia at ~3–4 kya and to the mountainous regions at ~5 kya. The data show that *S. japonicum* originated in the lake area of China, with the parasite radiating to Japan around 7 kya, to the mountainous region of China about 5 kya, and to the Philippines and Indonesia about 4 kya. The Taiwan parasite in this study, as the sister clade of human colonizing *S. japonicum,* diverged as a relatively independent isolate about 75 kya. This lineage might have lost the capacity to infect or was never able to infect humans during its evolutionary history. Indeed, it has been proposed that the genus *Schistosoma* arose in Asia from an avian schistosomatid, and this was followed by a host shift utilizing ungulates during the mid to late Miocene^15^. Interestingly, there is a recent report of a strain of *S. japonicum* from Changhua, Taiwan (KF279410) by Attwood *et al.*^16^, which might have migrated to Taiwan from mainland China. Combined with our data, we confirm that this Taiwan strain originated recently from the Lake region of mainland China ~5 kya (5.39 ± 0.81 kya) (Fig. S5). As a result, we assumed that there might be at least two genetically distinct isolates of *S. japonicum* in Taiwan, one being an ancient form we report here and the other is an isolate which may have arrived recently from mainland China^16^. However, as there has never been any report of an autochthonous case of *S. japonicum* from Taiwan, we consider the newly reported “Taiwan isolate” may instead be a “Chinese Lake isolate” which originated from a patient with a *S. japonicum* infection recently visiting or migrating from mainland China, although there is no detailed information provided for the sample analysed by Attwood *et al.*^16^.

Thus, we posed the question how could this parasite have radiated from the Lake area of China to other endemic areas? According to the life cycle of *S. japonicum*, there are two possibilities: the parasite was dispersed by its intermediate host or definitive hosts. However, almost no other species disperses as widely as *Homo sapiens* and, therefore, many human pathogens have achieved wide-spread distribution along with their human hosts^17^. Consequently, the spread of a pathogen is critically dependent on the extent of expansion of its human host. For example, *Mycobacterium tuberculosis* has experienced strong population expansion as a consequence of the recent human population increase^18^. Therefore, we hypothesized that there could be a similar correlation in expansion events between *S. japonicum* and its human host. Here, we investigate the demographic history of human being and *S. japonicum*. The reconstructed demographic history (Fig. 3 and Table S5) showed two peaks of population expansion, the first one happened at ~11 to 7 kya corresponded with two star-like lineages (Haplogroup B and A1; Fig. 2), and the second one happened ~5–2 kya, which might correlate with an expansion in the lineages A1b1, A1a2, A1a1b and A1a1a (Fig. S3). To investigate human migration and expansion in areas where *S. japonicum* was endemic during the transition to the agricultural era, we employed three human mtDNA lineages (i.e. B5, M7 and F) to construct BSPs for the human population. B5, M7 and F are three major human Haplogroups in the Southern part of East Asia with some sub-lineages having migrated to Southeast Asia^19^,^20^. Human BSPs showed two peaks of rapid N_e_ growth. One was a ten-fold increase at ~12–8 kya, followed by a subsequent three-fold expansion at ~5–3 kya (Fig. 3 and Table S5). The latter also coincided with the Neolithic dispersal of *S. japonicum*. To summarize, dispersal of the human-colonizing *S. japonicum*, including major expansions and migrations, occurred in Neolithic Time.

The transmission cycle of many pathogens can comprise a suite of species, each distributed according to its own ecological needs, thereby constraining the pathogen to the region where the host ranges intersect. Thus, the human BSPs correlate well with the *S. japonicum* BSPs (Fig. 3), indicating that *S. japonicum* expansion accompanied human activity, especially during the advent of the agriculture era. The data also imply that the migration of *S. japonicum* would be promoted by the migration of its human host over the past 10 kya. Indeed, modern humans have been reported to have migrated to South East Asia in the Neolithic period, with representative mtDNA haplogroups including M7b3, M7c3c and Y2^21^. Autosomal data also strongly support large demic movements of Austronesian speaking populations into Indonesia from ~4 kya^22^. This corresponds very well with the coalescence time (3–4 kya) of the Southeast Asian lineages of *S. japonicum*.

However, the data we present led us to pose another question. Why did this parasitic worm radiate from a lake region on the mainland of China to other endemic areas? Interestingly, when we reviewed the distribution of *S. japonicum*, we found that it coincided well with traditional rice-planting areas. This encouraged us to consider a possible relationship between the dawn of rice agriculture, and the transmission of schistosomiasis japonica. Beginning at about 12 kya, hunter-gatherer populations in the Fertile Crescent of West Asia began developing horticultural practices and commenced animal domestication^23^. In East Asia, agriculture started in the Yangtze and Yellow River Basins about 9 kya^23^. It is widely accepted that the Yangtze River is the original center of rice cultivation in China and other parts of Asia^24^,^25^. The planting of rice commenced about 8 kya in the middle and lower reaches of the Yangtze River, covering Zhejiang, Jiangsu, Jiangxi, Hunan and Hubei provinces, which are endemic areas for *S. japonicum* in the lake region. Rice-planting radiated to the southwest (endemic mountainous area of China), as well as to Southeast Asia (the Philippines and Indonesia) around 4 kya^26^, and it seems that the spread of *S. japonicum* followed the track of this agricultural practice. To achieve success in rice planting, people with appropriate skills and requisite tools, and a suitable environment are critical. Along with the spread of the practice of planting rice, subjects infected with *S. japonicum* would transmit the parasite from one location to another when they migrated to look for new cultivation areas. Moreover, the humid and warm environment required for successful rice cultivation is also highly favorable for breeding of *Oncomelania hupensis*, the intermediate host of *S*. *japonicum*, so that the parasite life cycle could be readily established and maintained in a new rice growing area. Considering that *O. hupensis* would already have been present in the agricultural era, as the divergence of these snails in the lake and mountainous regions of China occurred about 2–6 million years ago^27^ well before the parasites were introduced, their contribution to the life cycle of *S. japonicum* would be pivotal. As there are different subspecies of *O. hupensis* distributed in different endemic areas for schistosomiasis japonica e.g.^28^,^29^,^30^, and there are differences in compatibility between the snails and worms from different geographical locations^31^, we consider this separation of subspecies might not only have been an important reason for the survival of *S. japonicum* in new locations, but also led to the divergence of different geographically separated parasite populations.

The agricultural revolution, which involved the transition from hunting and gathering to settled agrarian societies, not only resulted in human migration but also led to a growth in human population size in Europe, Southeast Asia and sub-Saharan Africa over a period of 10 kya^32^. Our results lend support to the concept of the two population expansions of *S. japonicum* during the agriculture era, coinciding with human movement and population increase. For *S. japonicum*, many different species of definitive mammalian hosts and the key intermediate snail host (*O. hupensis*) characterize its zoonotic transmission cycle. The agriculture era not only resulted in expansion events of *H. sapiens*^33^, domestic and wild animals e.g.^34^,^35^, but also heralded the advent of rice-planting, increased land use and the introduction of irrigation along with human migration, which would have promoted the spread of *O. hupensis* and the transmission of schistosomiasis to new areas in East Asia. Indeed, we propose that the introduction of rice planting would have played a key role in promoting the transmission of schistosomiasis in East Asia.

## Methods

### Sample collection

119 *Schistosoma japonicum* worm samples in 13 locations were collected from Indonesia (Lindu lake, Sulawesi), Japan (Yamanashi strain maintained in the laboratory of Dr Sugiyama, National Institute of Infectious Diseases of Japan), the Philippines (Leyte), Chinese Taiwan (Changhua) and nine locations in mainland China (Table S1). In mainland China, two samples were collected from mountain regions (i.e. Eryuan County in Yunnan Province and Xichang City in Sichuan Province), while the other seven were obtained from locations in the lake regions (Duchang and Nanchang City in Jiangxi Province, Guichi and Tongling City in Anhui Province, Yueyang and Changde City in Hunan Province, and Shashi City in Hubei Province). From each location, 10 individual adult worms were sampled for sequencing as representatives of population diversity, with the exception that two individuals were collected from Japan. The protocol for worm collection is described in a previous study^36^.

### Preparation of genomic libraries and sequencing

Next generation sequencing (NGS) technology was applied to sequence the complete mitochondrial genomes of all the collected samples of *S. japonicum*. We first amplified the complete mitochondrial DNA (mtDNA) genomes using 13 PCRs, which cover the whole mtDNA genome. These 13 overlapping products were then mixed in roughly equal amounts after determining the concentration of each amplicon. Then, fragment libraries were prepared using the optimized protocol provided by Illumina and published^37^. Briefly, the complete mtDNA genome of each sample was sheared with DNase I and the sheared fragments were purified and concentrated using a QIAquick PCR purification spin column (QIAgen Inc.). T4 DNA polymerase, T4 phosphonucleotide kinase and the Klenow fragment of *Escherichia coli* DNA polymerase were used to fill 5′ overhangs and remove 3′ overhangs of sheared fragments and then were added A-residues at the 3′ terminal sides using dATP and Klenow (3′–5′exonulcease). Adaptors containing unique barcode sequences were then ligated to the fragments. We harvested fragments ranging from 200 bp to 250 bp through an agarose electrophoresis platform, the products were isolated using QIAgen MiniElute gel extraction spin columns and then each sample was amplified using standard Illumina primers and running 15 PCR cycles. After these libraries were re-purified, we quantified the DNA concentration of all samples and 30 ng of each were pooled together. The oligonucleotide mix was finally sequenced on an Illumina HiSeq 2000 by BGI, China.

### Whole mtDNA sequence assembly

Original sequencing reads were exported to Fastq files, and then bwa v0.6.2^38^ was used to align reads to an existing *S. japonicum* mitochondrial genome reference sequence (Genbank accession: NC_002544) to generate binary sequence alignment/map (BAM) files of the mtDNA genomes^39^. Duplicate reads were removed by MarkDuplicates, implemented in Picard v1.82 (http://picard.sourceforge.net) and the mtDNA sequences were locally realigned by GATK v1.2.59^40^. Pileup files were generated by SAMtools v1.0.18^39^. Consensus sequences were then obtained based on the pileup files, and indels were checked manually afterwards. Variations for haploid were called according to the criteria used in Zheng *et al.*^41^. All the 119 sequences obtained were deposited in Genbank (KU196299-KU196417).

### Phylogenetic analysis and time estimation

As an outgroup to *S. japonicum*, the mt genome sequence of *S. mekongi* (Genbank accession: NC_002529) was selected and aligned to *S. japonicum* sequences considering gene rearrangement^15^. The phylogeny of 119 *S. japonicum* mt genome sequences and one *S. mekongi* mt genome sequence was inferred by Mrbayes v3.2.1^42^ with the HKY+I+G model. 10^6^ generations were performed with 4 chains (i.e. 1 cold chain and 3 hot chains) and the first 7,000 generations were regarded as burn-in. Moreover, the PhyML v3.0^43^ with HKY+G model and Network v4.6.1.1 (http://www.fluxus-engineering.com/sharenet_rn.htm) were applied to generate topologies. Haplogroups were defined according to the topology and assigned to each sample.

For the calibration point, we assumed that the divergent time between *S. japonicum* and *S. mekongi* was 3.8 million years ago (MYA), according to a recent study^14^. The phylogenetic tree was used to examine the assumption of a molecular clock under the HKY+G mutation model. The null hypothesis of a molecular clock cannot be rejected (P = 1.00) using the PAML package v4.7^44^. We employed two strategies to estimate the ages of *S. japonicum* haplogroups, i.e., the maximum likelihood (ML) method via PAML package v4.7 and the Bayesian method via BEAST v1.8^45^. In ML analysis, three sequence partitioning methods were performed with the HKY+G model. First, we used the complete mtDNA genome sequences, which were partitioned into 6 regions (i.e. the first, second, and third positions of the codons, tRNA, rRNA and non-coding regions). Second, we used protein coding regions only (i.e. partitioned in three positions of codons), considering that historical gene re-arrangements between *S. japonicum* and *S. mekongi* might cause alignment problems with whole sequences. Third, we used the third position of codons only for the reason that most variants on the third codon were synonymous mutations. Natural selection on mtDNA is thought to have an effect on the mutation rate of the whole genome and time estimation. However, synonymous mutation rates are regarded as neutral and free from negative selection. In Bayesian analysis, whole mitochondrial sequences were employed and partitioned into 6 regions as with the ML analysis. We ran 10^8^ iterations, with samples drawn every 5,000 steps and the first 10^7^ iterations considered burn-in. A strict clock was selected in all analysis.

We reconstructed the historical demographic variation of 109 human-infecting *S. japonicum* sequences via Bayesian skyline plots (BSPs) implemented in BEAST v1.8. Tracer v1.5 was used to visualize the results and to construct the BSPs.

### Human mtDNA data

The 435 complete human mtDNA sequences of Haplogroups B5, M7 and F were used to construct the BSPs for the human population. B5, M7 and F are three major human Haplogroups in the Southern part of East Asia with some sub-lineages having migrated to Southeast Asia^19^,^20^. The 435 human mtDNA sequences were from 3 random population data sets, of which 175 sequences were from the 1000 Genomes Project^41^, 68 from the Human Genome Diversity Cell Line Panel (HGDP-CEPH, Genbank: KJ445738- KJ446778 and KP240908-KP240930) and 253 newly generated sequence data (Genbank: KP240655-KP240907). The sequencing and variant calling method have been described previously^46^. The parameters for BSP construction were as we have described previously^41^,^47^. Population growth rates were calculated from the BSP using the method described in Gignoux *et al.*^32^.

## Additional Information

**How to cite this article**: Yin, M. *et al.* Co-dispersal of the blood fluke *Schistosoma japonicum* and *Homo sapiens* in the Neolithic Age. *Sci. Rep.* **5**, 18058; doi: 10.1038/srep18058 (2015).

## Supplementary Material

Supplementary Information
