# Supplementary material for: Co-dispersal of the blood fluke Schistosoma japonicum and Homo sapiens in the Neolithic Age
Source: Sci Rep. 2015 Dec 21;5:18058. doi: 10.1038/srep18058 (PMC4685303; doi:10.1038/srep18058)
Supplement: Supplementary Information [file srep18058-s1.pdf]

Co-dispersal of the blood fluke *Schistosoma japonicum* and *Homo sapiens* in the Neolithic Age

Mingbo Yin[1#], Hong-Xiang Zheng[1#], Jing Su [1], Zheng Feng[2], Donald P. McManus[3], Xiaonong Zhou[2], Li Jin[1,4], Wei Hu[1,2*]

1 State Key Laboratory of Genetic Engineering and Ministry of Education Key Laboratory of Contemporary Anthropology, Collaborative Innovation Center for Genetics and Development, School of Life Sciences, Fudan University, Shanghai, 200438, China
2 National Institute of Parasitic Diseases, Chinese Center for Disease Control and Prevention, Key Laboratory of Parasite and Vector Biology of the Chinese Ministry of Health, WHO Collaborating Center for Malaria, Schistosomiasis and Filariasis, Shanghai, 200025, China
3 QIMR Berghofer Medical Research Institute, Brisbane, Queensland 4006, Australia
4 Chinese Academy of Sciences Key Laboratory of Computational Biology, CAS-MPG Partner Institute for Computational Biology, SIBS, CAS, Shanghai, 200021, China
# Equal contribution author
* Corresponding author: Wei Hu; e-mail: huw@fudan.edu.cn; phone: +86 (0)21 5163 0662; fax: +86 (0) 21 5163 0663

Table S1. Detailed information of *S. japonicum* samples used in the analysis.

Population	N	π	θ	Latitude	Longitude	Infection[a]	Detailed population location
AHGC	10	0.00462	0.00419	30.67	117.45	0.14%	Anhui Province, Guichi Country, Minsheng Village
AHTL	9	0.00403	0.00397	30.94	117.76	3.25%	Anhui Province, Tongling Country, Laozhou Island, Guanghui Village
HBSS	10	0.0041	0.00306	30.32	112.35	<0.01%	Hubei Province, Shashi City, Maling Village
HNYY	10	0.00492	0.00673	29.34	113.07	0.02%	Hunan Province, Yueyang City, Laogang Village
HNCD	10	0.00349	0.00389	28.94	112.16	0.93%	Hunan Province, Changde City, Wuyi Village
JXDC	9	0.00475	0.00462	29.2	116.5	6.50%	Jiangxi Province, Duchang Country, Tangmei Village
JXNC	10	0.00553	0.00663	28.21	116.16	n.a.	Jiangxi Province, Nanchang City
Lake	68	0.00491	0.00939	n.a.	n.a.	n.a.	n.a.
SCXC	9	0.00103	0.00107	27.5	102.2	0.14%	Sichuan Province, Xichang City, Daxing Township, Shian and Jianxin Villages combined
YNEY	10	0.00042	0.00043	26.14	99.95	n.a.	Yunnan Province, Dali City, Eryuan Country
Mountain	19	0.00108	0.00132	n.a.	n.a.	n.a.	n.a.
TW	10	0	n.a.	23.85	120.92	n.a.	Taiwan
IN	10	0	n.a.	-2.2	120.1	n.a.	Indonesia
PH	10	0	n.a.	14.69	121.37	n.a.	Philippines
JP	2	0	n.a.	36.5	138.3	n.a.	Japan
ALL	119	0.00657	0.01171	n.a.	n.a.	n.a.	n.a.

[a] Infection rate in local snails

Table S2. Distribution of mutations in the S. japonicum mtDNA genome.

	rRNA	tRNA	Protein coding region				noncoding	total
			all	1st	2nd	3rd		
Length (bp)	1748	1468	10076	3346	3384	3346	795	14087
Unvaried sites	1688	1412	9437	3189	3324	2924	672	13209
Number of varied sites	60	56	639	157	60	422	123	878
Proportion of varied sites	0.0343	0.0381	0.0634	0.0469	0.0177	0.1261	0.1547	0.0623
Sites with a single hit	51	50	528	137	55	336	90	719
Sites with two hits	7	6	95	19	4	72	27	135
Sites with three or more hits	2	0	16	1	1	14	6	24
Indels	6	4	0	0	0	0	20	30
Substitution	65	58	773	178	66	529	159	1055
Substitution per base pair	0.0372	0.0395	0.0767	0.0532	0.0195	0.1581	0.2	0.0749
Transitions	54	50	650	158	55	437	133	887
Transversions	11	8	123	20	11	92	26	168
Transition/transversion ratio	4.91	6.25	5.28	7.9	5	4.75	5.115	5.28

Table S3. Age estimates for important haplogroups in *S. japonicum*.

		Maximum likelihood (kya)			Bayesian (kya)
Node	*n*	complete sequence	protein coding	3rd position of codon	complete sequence
A'B'C'D	119	75.43 ± 9.58	75.11 ± 12.87	89.86 ± 16.38	75.45 ± 11.9
A'B'C	109	21.76 ± 2.45	20.55 ± 3.28	22.77 ± 4.58	22.95 ± 3.77
A'B	101	21.55 ± 2.44	20.55 ± 2.88	22.77 ± 4.22	22.89 ± 3.77
C	8	15.4 ± 2.08	13.95 ± 2.45	15.39 ± 3.39	14.87 ± 2.61
B	23	9.25 ± 1.17	7.66 ± 1.18	9.22 ± 1.78	9.56 ± 1.64
B1	12	7.01 ± 1.04	5.99 ± 1	6.94 ± 1.43	7.16 ± 1.29
B2	2	6.65 ± 1.17	5.61 ± 1.17	6.95 ± 1.69	5.95 ± 1.25
B3	4	8.73 ± 1.27	6.62 ± 1.52	7.75 ± 2.19	7.96 ± 1.6
B4	4	1.87 ± 2.29	0.99 ± 2.37	0.54 ± 5.59	1.97 ± 0.76
A	77	13.37 ± 1.7	12.27 ± 1.83	12.81 ± 3.12	13.82 ± 2.31
A2	3	5.48 ± 1.54	5.11 ± 1.62	3.33 ± 2.17	5.53 ± 1.33
A1	73	10.68 ± 1.21	10.13 ± 1.38	11.14 ± 2.12	10.94 ± 1.78
A1a	48	7.05 ± 1.15	6.5 ± 1.36	7.14 ± 2.42	7.83 ± 1.38
A1a2	29	5.82 ± 1.12	5.24 ± 1.42	6.15 ± 2.42	6.35 ± 1.16
A1a2a	24	3.41 ± 1.33	2.89 ± 2.26	3.19 ± 3.5	3.84 ± 0.83
A1a1	19	5.42 ± 1.03	4.84 ± 1.16	5.3 ± 2.75	5.97 ± 1.11
A1a1b	10	2.41 ± 0.96	1.58 ± 0.94	1.69 ± 1.41	3.05 ± 0.79
A1a1a	9	4.21 ± 0.98	4.33 ± 1.09	4.55 ± 2.81	4.75 ± 0.94

Table S4. Mutation rates estimated based on ML and Bayesian methods.

	Maximum likelihood ($\times 10^{-8}$/site/year)			Bayesian ($\times 10^{-8}$/site/year)
	complete sequence	protein coding	3rd position of codon	complete sequence
rRNA	6.67 ± 1.13	-	-	7.17 ± 1.48
tRNA	8.52 ± 1.33	-	-	7.92 ± 1.59
non-coding	42.10 ± 5.87	-	-	40.59 ± 7.54
1st codon	10.39 ± 1.23	11.20 ± 1.54	-	10.84 ± 1.81
2nd codon	4.17 ± 0.60	4.30 ± 0.69	-	3.89± 0.72
3rd codon	33.33 ± 3.62	35.70 ± 4.79	30.70 ± 5.04	30.95 ± 4.85

Note: Mutation rate estimates showed that the non-coding region evolved fastest for free of selective constraints while rRNA and tRNA were relatively conservative. In the protein coding region, the third position showed the highest rate as was evident for the non-coding region, while the second position evolved slowest due to its high effect on amino acid change.

Table S5. Population growth rates calculated from Bayesian Skyline Plots.

Datasets	Era	Growth began (kya)	95% CI for start of growth	Population growth rate (%)	Fastest growth time (kya)	Maximum growth rate by interval (%)	Peak interval (kya)	Peak growth rate (%)
S. japonicum	Neolithic Time	5.8	4.6-7.0	0.01	2.2-4.4	0.02	3.4	0.024
	Upper Paleolithic Time	11.4	10.4-12.8	0.005	8.2-10.2	0.015	9.2	0.016
Human	Neolithic Time	5.6	4.6-7.0	0.005	3.4-5.2	0.027	4.6	0.033
	Upper Paleolithic Time	14	12.2-16.6	0.002	8.4-12.2	0.027	10.8	0.038

Figure S1. MtDNA coverage distribution of the 119 *S. japonicum* samples used in the analysis. Note: Average coverage (black line) with 5% and 95% quantile coverage (dashed lines) for each mtDNA genome assembled in the study.

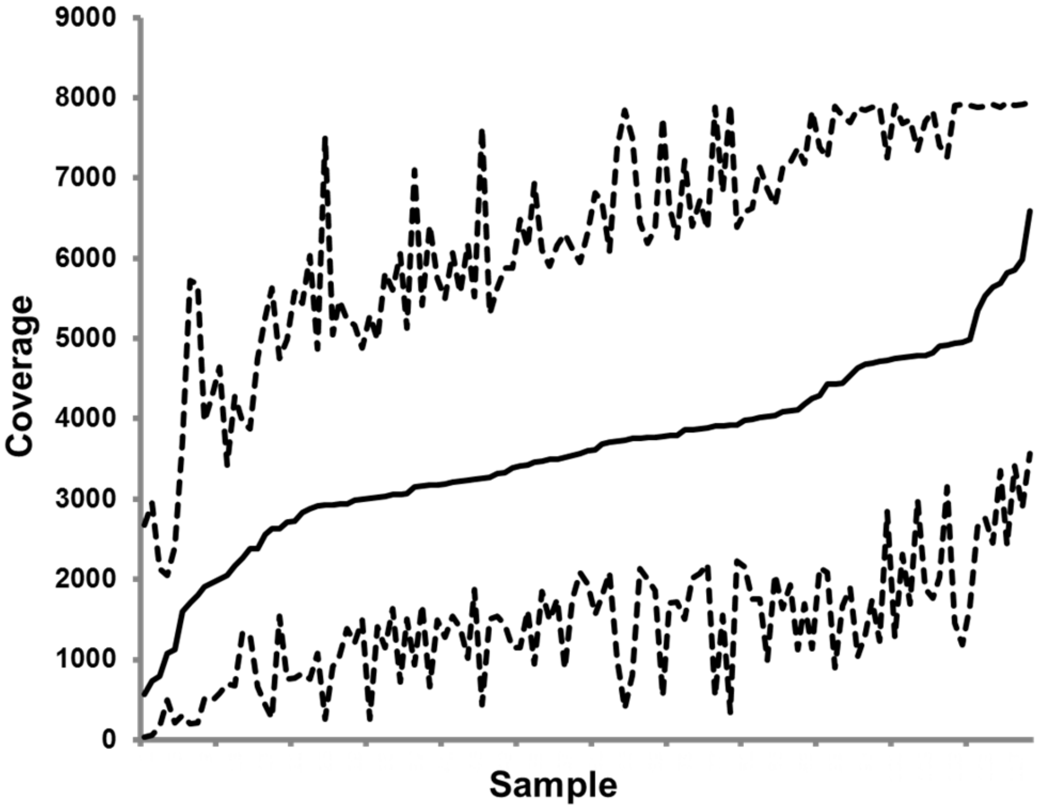

Figure S2. Variation distribution across the mtDNA genome of *S. japonicum*.

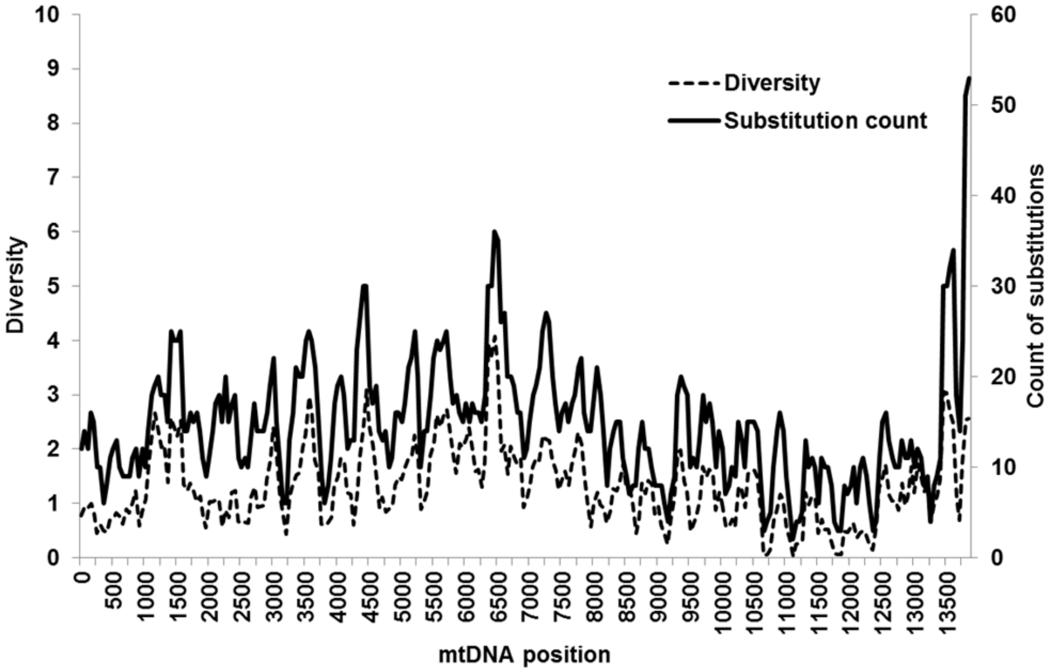

Note: The diversity and substitution were counted and calculated every sliding window of 200 bp (step size = 50 bp) along the entire mtDNA genome.

Figure S3. Detailed phylogenetic tree based on the mtDNA sequences of 119 *S. japonicum* samples. Every mutation is denoted to each haplogroup. Insertions are represented as '.'. Deletions were added as 'd' at the end. Blue denotes non-coding mutations. Red indicates synonymous mutations while Black means nonsynonymous mutations. Green indicates tRNA and rRNA variants. Refer to Table S1 for the abbreviations of each sample. (See supporting file 1)

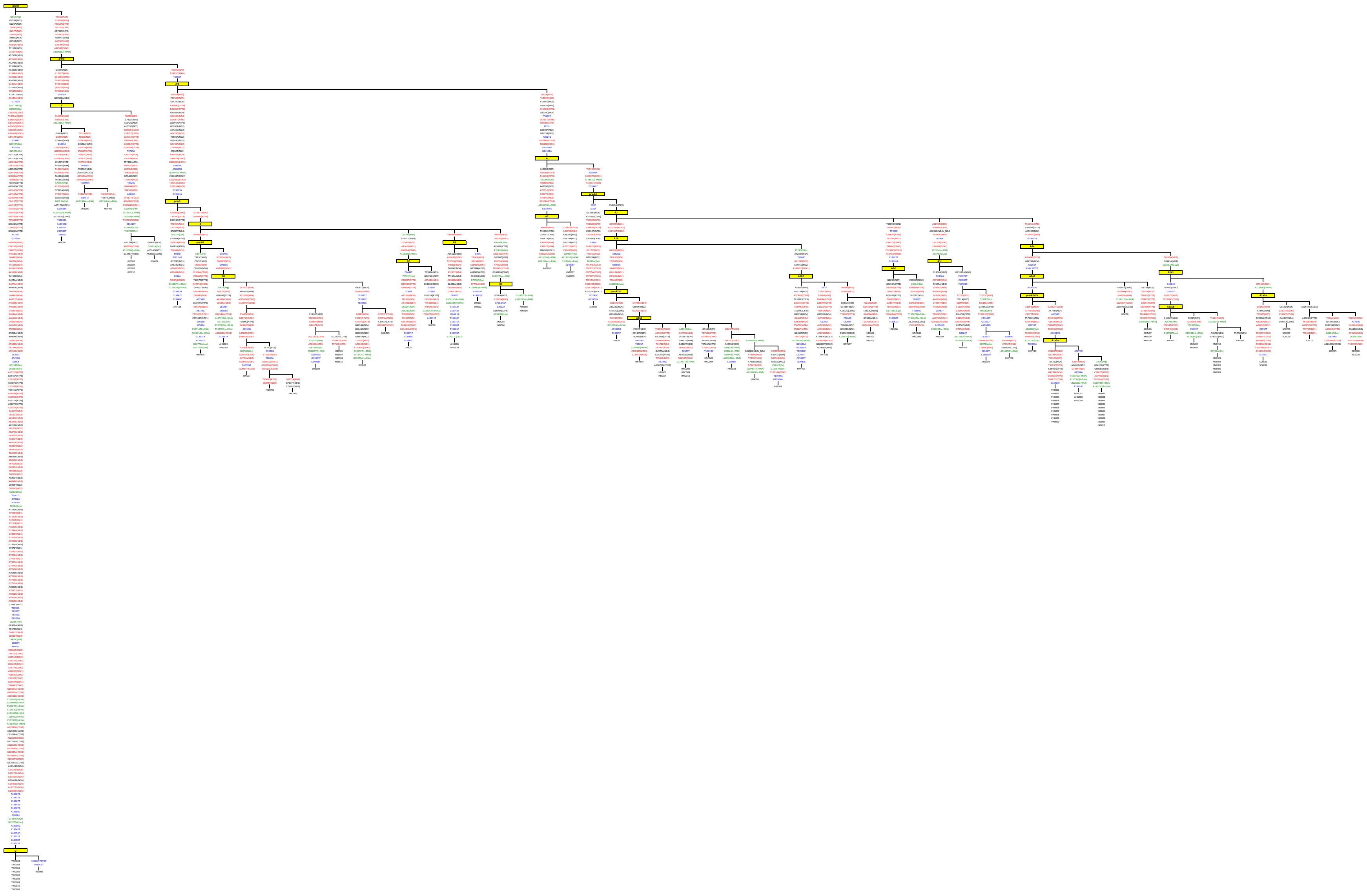

Figure S4. Time estimates of *S. japonicum* haplogroups.

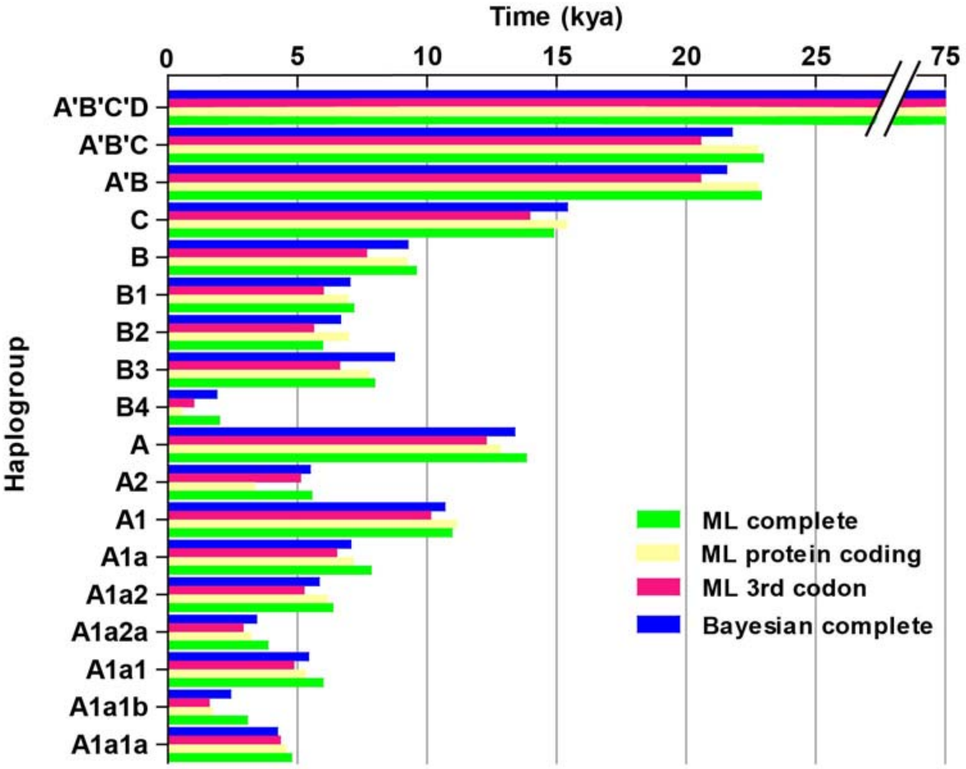

Figure S5 Phylogenetic tree of 120 complete *Schistosoma japonicum* mt genomes, based on Mrbayes. The red ID indicates the sequences from Attwood *et al* (2015). For the abbreviations of each sample, refer to table S1.

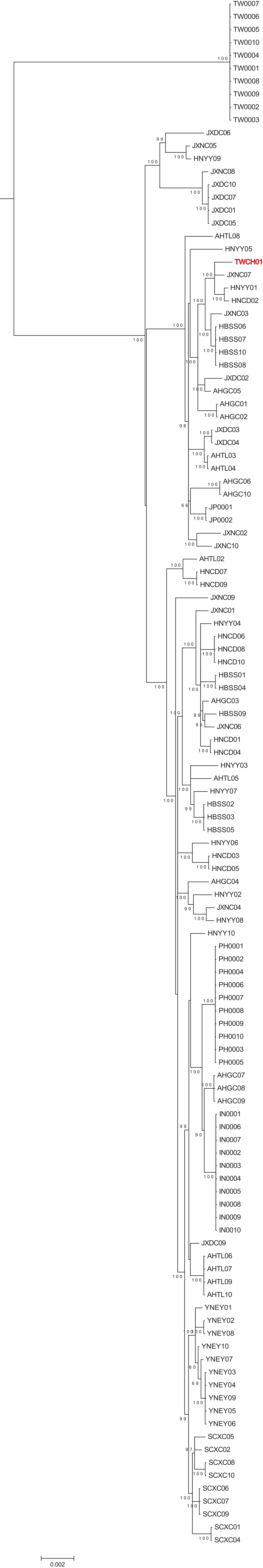
TW0007
TW0006
TW0005
TW0010
TW0004
TW0001
TW0008
TW0009
TW0002
TW0003
JXDC06
JXNC05
HNYY09
JXNC08
JXDC10
JXDC07
JXDC01
JXDC05
AHTL08
HNYY05
TWCH01
JXNC07
HNYY01
HNCD02
JXNC03
HBSS06
HBSS07
HBSS10
HBSS08
JXDC02
AHGC05
AHGC01
AHGC02
JXDC03
JXDC04
AHTL03
AHTL04
AHGC06
AHGC10
JP0001
JP0002
JXNC02
JXNC10
AHTL02
HNCD07
HNCD09
JXNC09
JXNC01
HNYY04
HNCD06
HNCD08
HNCD10
HBSS01
HBSS04
AHGC03
HBSS09
JXNC06
HNCD01
HNCD04
HNYY03
AHTL05
HNYY07
HBSS02
HBSS03
HBSS05
HNYY06
HNCD03
HNCD05
AHGC04
HNYY02
JXNC04
HNYY08
HNYY10
PH0001
PH0002
PH0004
PH0006
PH0007
PH0008
PH0009
PH0010
PH0003
PH0005
AHGC07
AHGC08
AHGC09
IN0001
IN0006
IN0007
IN0002
IN0003
IN0004
IN0005
IN0008
IN0009
IN0010
JXDC09
AHTL06
AHTL07
AHTL09
AHTL10
YNEY01
YNEY02
YNEY08
YNEY10
YNEY07
YNEY03
YNEY04
YNEY09
YNEY05
YNEY06
SCXC05
SCXC02
SCXC08
SCXC10
SCXC06
SCXC07
SCXC09
SCXC01
SCXC04
0.002

Supporting information:

>ref

CTGGTAATCTTGTAAGAGGGTTGTGTTTCGGCCACAATTATGTAGAGTTTATTGTTTCTACTACCAGTGTTTTT
GCTGATGTTGTTAGGTTGAGTGGTATTTTTTTTTTTTTTTTGGTTTTTTGTTTTAAATAATATAGATTTTGGTGG
TTGAGTTAAGTTTAATGTTGGTGGTTATTTGGTGTGTGATGTTTTTGGTTTTTCTTATAATAAAGAGTATAGTTC
TTTAGTTATTTTTATGTTGATGATTTGTACTTATATATCTGTTATGTATAGTTTTCATTATTTTTCGTTAAGGAATG
TCTCTTGGTTACTTTCTTATATAATGGTTAGTTTTTCTTTGGTTATGATTATTTTAGTAATGACTAATAGTTTTTTA
GTTAGGTTAATAATGTGAGAGTATCTTGGTTTGATTAGTTATATTCTTATATGTTTTTATGATAATAGTTCTTCTTT
GCGTGGTGCTATGGTAACGTTAGTTTCTTCTCGGTTTGGTGACGTTTGTTTTTTTATTTTGGTTTCTTTATATTA
TATTGGTGGTTGTGCTTATGTAAGTTTGTTAGGGTTAGTAGTCTTTGTGATTGTGTCTACTAAGAGTGCTTTGT
TTCCTTTTATTTCTTGATTGTTAGAAGCTATGCGTGCTCCTACTCCAGTAAGTTCTTTAGTTCATTCGTCAACTT
TGGTTGCTTCTGGTGTCTGAATGGTTGGTAGTTATTATTATTTAATTGGGTTGGGAAAAAATAGTTATTTAGTT
TATTTTTTTCAGTGTTGTTGTTTTTTGACAATAGTGATAACTTCTGTTTGTTCTTTATATTATAGTGATGTTAAGC
AGTTAGTTGCTTTGTCTACTAGTAAAAATATTTCTTGGGTTTTTTTAATTTTGTCTATAGGTGATTATGTATTGG
CATTTGTTCAGTTATTGGTTCATGCTGTTAGAAAGTGTTTATTATTCACTTTGGTTGGTGATTATATAAGTTCAA
GTTATGGTGGTCAAGATAAGAATCAAATTAATACTAAAATTTATGGTAAAGTGTTTGGTTTAATTTATTTATTTG
TGTTGTTGGTTGGTTTATCTGGTTTTCCATTTATGGGTTTATATTTTAGAAAGCATGTTTTTTTAGATAGGTATA
CGAATATAGGGTTTTTTAGTATATTGTTTTTGTTTTTAGTTATTTGTGGATTTATTATGTCTTGTGCCTACAGTGT
TCGATTGTTTATTATATTGTTTGGCTATTTGTCGTTTAGTGTGTATTCACTTCGTGTAGATTTTAATATG ATAGGT
TTAGTTGTTATGATTAGTTCAATAGTTGGTAAATATATGTGCTTTTTAATTTATAGGCCATATAGATTTTTTAGTT
TTTTAAGTTTTTTTGTTTTATTTAGATTATTATTTGGAGTTGTAGTAGGTGTAGTGGTTGGTTGTAATTATATTAA
ATATCGTAGAGATTGGTTAGGTAAATTGTTTGGGGTTGATTTTTTGATTGATATGTATAATCGGTATGTTTTGTA
TATATGAAATTATTTATTTTTATGTTTGTTTCGTTGAGAAATATATATGTTTGAGTTTCTAAATGATTGTGTTTCTT
TATTTTTGTTTAGTTATTTATTTAAGGTTACTGTTTTAGTAGTTAGTGTGTTTTTTTCTTTATTTATTTTGTTTTTT
TAGTAAATTATTTTAAGATTAGTAGTGGTTTTAATTTGTGGTAATATAACGAGTGTTATAAGTTTTATTATTCTTT
CTTTCCAAGTAGGAGATCTCAGTTGTTTGAGCCGTTATATATAGTTATG AGTTTATTTTCATTAGTAAAATTAGT
GTTGTTGTTTTTCATATTACCTTGTATATTTTTTTTTCATCCTTTTATTTTGGTGTTAATATTGTGTTTATGATTTTA
TTTGTTCAATCGTTATGTTAGATGGGAGTTTGTAAATATAACTACAGATCGTATAGTTGGATTTTGATTGTTTTT
AGTGAGTGAAATAATTGTTTTTGCTACTCTTTTGTTTACTTGTTTGTGATTTCAAGATTATTATAGTAAGCCGAT
TGCATATGCTTATGGTGCTCCAATGGTTGAAAGTTGGTTGTTGATATCTTCTTCTTTTTTTATGACTTCATATCG
AGGTTTAATTAATACTAAGTGATGTCATTTATTTTTAAATTGGAGGATTATCTTTTCTTTTTTTTTTATGATAACT
GCTGTTTTAGAGGTTATAAGGAGTGGTGTTAGTAGATTATTTAATCCGCATGCTGCGGCTTGTTATATGACTAT
AGGTTTGCATTTTATACACGTTGTAATAGGTACTGTAGGATTGACTCAATTGGATTATTATTTTAGATTTGATGT
TGTTCGTCGTTATAGATGGATGATTGTTGTGTATTGGCATTTTGTTGATTATGTATGGTTGGTTGTTTTTACCAT
TGTGTATTTGTTAGTTTAAGTTAGTTGGTTACGGTTAGCCAGGTGTGATGTGCATATAGATTTTTCGTTTCTGT
GGAGTTAGATTGTTTTTAACGGTTAGCATAAGTTTAGTTTTATGTAGGTTAAAATAAACCGTTAGTTTGTGGTA
CTAAAGATTTTATGTTTTGAACTAAAATATTGGATGTTGAAAGTCATTCGTCTTAATTTGGTTGATTTGCCTACT
AGGTTGTCTCTTAAATATTTTTGGTGTGTTGGTTTTGTGTTGAGTATTTTTATGGTTATTCAAGTAGTTTCTGG
AATAATTTTGTCATTATTTTATGATGTTTTAGGTAAATTTTCTTTATTAATGATGTGGACTGATGATAGAATTTGA
TGTTGATTTATTCGTTACACGCATATATGATGTGTTAGTGTAGTGTTTTTTCTTATCTATACTCATATGGGTCGAA
GGTTGTATTATGGGAGGTATCGTTTGCTTGGCGTTTGAAAAGTTGGGTTTTTGATTTATATTCTTGTGATGATT
GAGGCTTTTTTAGGTTATGTTTTACCATGACATCAAATGTCTTATTGAGCTGCTACTGTATTGACTTCTATAGTT
TTAAGTATTCCTGTTATTGGTGTTAGGTTGTATAATTATATTATTGGTGGATTTTCTGTTACTTTGGTTGATACAC
TTATTCGTGTATTTCCTGTTCATATAAGTTTGGGGTTTGTTTTGTTAGGATTAATTATTTTGCATTTATTTTATTT

GCACTTGTATGGTTCAAGTAGTCCATTGTTTATTTATAAAAATTATAGTGATTGTATTTATTTTCATAGTTATTATT
CTATAAAGGATTTGTTTGTTTTTATGATAGTTATTTCTTTAGTTAAATTTATGATAATGATTGATCCAAAAATGAT
ATTAGATGTTGAAGCTTTTGTTGTTGCCAATCCTTTGGTGACTCCAGAATCTATAAAGCCTGAGTGATATTTTT
TGCTGTTTTATGCTATGCTACGAAGAGTAGACTCAAAGATAGGTGGTTTAATATTAGTAATTTCATTTTTGGTT
GTTTTGTGATTGCCAACAAGTAAATTTAGATCTATATATGTTTTAGATCGTCAGATTTTTTTTTGACTTATGGCT
AGTTTATTTTTTATGCTTAGTTATTTTGGTGGTTGTCATGCGGAGTATCCTTATATTTATGTGAGTAAGGTTATA
AGGTTTTTTATGTTAGTTTTATTAGTTACTTATAAGGTGTTGTGGATTATTCCATTGGGTGTCGATAGTGATTAC
GTAATTTATCAAGTTTGATAGATAGATTATATCTATGTTTTTTTAATTTAAATAATGTTATTTGTCATTTTATTACT
TGGAGGTGGTTTGATGTTAATTAGACTGGTAATATGTAGTCACTATTTGTTTAAATATTTAATAGTTCTTGAGA
GTTATAAAGTTTTATTATTATTAATTTGTTTGTTAGTTAAAGTTCAGGATTGTCAAGTTGTTTTCATTTCGATGAT
GGCTTTGTTTGTTTTAGAGGCTTCAATAATGTTAATTGTTGTTGGTATAAGAATATGTCACGGTTCACTTCGTG
TTTCAGGAGGTTTATAACTGGATTTGTAGTCACTTTTATATTTTTACTTATCTTTTATTCTAGTATATGGGTTTCA
GATAGTTCTATGGTAATGGTGGGCGTTAAGTATTATTTATGTGATGGTTTAGTTATAATTGATACATTATCATGTT
TAATGATTTTTTTAACTTCTATAATATGACTTGTATTGTGGTTAGTTGGTTCTAAGGATATAGTTTTATTTATTAGT
GTTTTTAGTGCTATGATTACATATGTTGTATCTAAATCTTTAGTATTTTGGTTTTTCTATGAGCTTTCAATTATTAG
GGCTTTATATATGTTAATTGTTGGTAGTCCTTATCCAGAACGTTATATTTCTAGGTGATATTTTGGTGGGTATATA
CTATTAAGTAGTGTTCCGTTGCTATTGGGTATTTGTTTTATTGGTTTGAATAGTGGTAGGTTTAAAGTGATTTT
GTGAGATAAGGGGGATATGTGTGATTCTTATGGTGCTTTTTTGCTCATTATAGTAATGTTTTTGACTAAGATTC
CTGTTTTTCCATTTCATGGTTGATTACCACTAGTTCATGCTGAAGCTAGGAGTCCTGTCAGTATAATATTAAGT
GGTTATATAATGAAGTTGGGTTTAGTTGGGTTAGTGCGTTTATGTGGATGACTATTAATTGATTATATATATTATT
TTTCTACTTTTTTATTATGCTATAGAGTAGTTTATTTAGTTGCAGCTGTGTTTGAATGTGATTCTAAGCGTTGAT
TGGCTTATCTTAGGTTGTCTCACATATTGATTGGTGTATGTATATTACTAACTTCTACGTATTGTGGTGATTATCT
TGCTTTCATATATTGTTTAGGTCACGGATTATCTGTTGCTTTATTGTTTATGGTTATATGATTTGGTTATGAGATT
AGTGGTTCTCGTAAATGGGGTATACTTGTCAAGATATTTGGTGGTGGTTTAATTATGCATTTTATTATGGGGTT
TGTGTTTTTAAAAGTTTGTGGGTTTCCACCAGCCTTGCAGTTTTTTGGTGAGTTATGATTGGTTATAAAATATA
TTACGTTGGGTGATATAATATCATTATTATTAGTATCTATTTACATATTTAGCGGTAGAATAATAGGTTTTATTATT
TATGGTTTAGTAATATGTTCTCCTATTAATACAAGATATGAGTATAGTGGTGGATTGGATAAATTTTTGTTTTGTA
TTATGTATTTAAGTTTATTAAATTTAATTTTGTTTATAGTATTATAGCTTAAGATATATGACTATTATTAAAATGTAA
GAAGCTAAGTGTAATTTATTGCATGTTCTTGTTTTGGTCAGGATGGGGGTTGGGTGCCAGTTTCTATTTTATTT
AATTATTAGGTTTGTGAATTAAAGCCAAAATTTTTGATTTTAGTTGCGATTATTCCTTTTAGCTTAATATTGAGA
GTGTCAGTTTGAAGGTCTGGAGGTATATTTTATATAGAGGTGGATATTAAGTTAATAAAAACTGTATGATTCAT
GTTCATGTATTGTGCGGTAATGCACATATCCTTATGTTATCTGGGTTATGGTTTACTAGATTTTTGGTTAAGCTG
TCTAGTTATTTAAAGGTGATGAAGATTGAAATGTGGTATAGCTTATCTTTGTTTATACTGATTTTTGTATTTGCC
ATAGTTCGTTTTCCATATGTTTACGGGATGTTTGATTATGGGTTATGTTTAATAGTGTTAATTATGCCATTGTTTA
TCTCCTTGTTTTTTAGGCGAATGTTTTGTGCGTTTGGTTTGTTTGTTTCAAGGTTTATACCAGTTGGTTGTCCG
ATATACTTAGCACCGTTTGTTTGTATAATAGAGTTAATCAGGTTTGTTATACGTCCAGTCGTTTTATTGGTCCGT
CCGTTTGTAAAAATAAGTGCTGGTGTATATTTTGGTGTATTTGTTGGTAATTTGTGCTTTGCTATTAATTATTTTA
TTTTTGTAATTTTTTTTGTGCTGTTTGTTTATGAGATTTTTGTTGCATTAATGCATTGATTTATAGTACAAGAGAT
ACTTAAGTTTTCTGTCGATCATTAATTTAAGTTTAGACATATGCTTTATTCATTTTTGGCTATTACTATTTTGTTG
TCATTATGTGTAACTCTGGTATTTTCAGGTGACTTATTGACTTTTTGATTATTATTAGAGTTATGTAGGATAGTA
GTTATTCCATGTTTTTATTGGAATGATAATATTAGAGCCTTGAGTCAGGTTGATGGTCTGCTTTATTATTTACTT
GCAACCAGTATATCATCTTCATTGATTTTGGTTGGTATTTTATTTCCTGGTATTTTTTTTTTTTTTTTTTTTTGGTT
TTTTTTTAAAGTTCGGTGTGTTCCCTTTTATTTTTTGGGTTTATCAGGTTTTTACATCAAGTAAGAGTTGAATA
ATTTGTTGATGTATTTCTACTGTTTTAAAGTTTCCTGTTTTATATATTAGTTTTTTTGTGGGTCAATTTAATATATC

GTTGGCTATTATTCTGTCTAGATTGGGTATATTAATTTCCGGTGTGTTGATTTGGGTTAATAGTATTAATTGGTT
TGCTGTTTGATGTTATATGATGGTTTCTTCTAGTAAAGTGATAGTTTGTTTAAGTGTTGATTGTTCGTTTTTTAA
TTTGCTTATAGTTTATTTAGTTTATTTTATTTGAAGATCTGGTGTTATATTTTATTTAAGTAGCTTTTATGGTGGT
GTTTTTGGATATGTAGTTTGGTTAATAGCTATTCCTTTGAGTTTTGCTCTTTATTATAAGATTTATGTTAGTTATT
TGTTGATTGGATTAGGGTGAGTATTTGTTTTTTTTTGAGTATTATATAGTTTTTTAGAGCAGTTTTATTTATTTA
AGTGGTTAGTTAGTAGTACTGTACCAAAGAGTACTTGATGAGGACGTGGGAAGATCTTGTTTTAGTTTTAAC
AGTATATTTTAAGTATAGAATGTCTGTTCTGCAAACAGATGGTGAAGATAATTTTTCTATTGTTATTTTTTTCTTT
ATTGATTACGTGCTTAGTTTATTTTAGAATTGGAGTTTGTCAGGCTTTAGGAATCTCTTTTGGATAGTGCGTTA
ATGAAAATAAGTATGAATATATGAAACTTTTTAGTTTGGTTAGAGGGTTTGTTGGTGGTTTTGTTTTTAGTAGC
ATTTTTTATATTAAGGGAGCGTAAGGTTTTAGGGTATATTCAATTGCGTAAGGGTCCGAATAAGGTTGGTTTA
GTTGGACTATTTCAGAGTTTTGCTGATTTTATTAAGTTATTAAGTAAGTATAAGATCGATGGTTATACTGTTCAT
AGTTGGTTTAGATGATTTGGGTGTGCTATATTATTAATATGTTCTTCTATTGGTGTATTTATTTATGCTAGTGACT
GTGGGGGTTTGTATTATAAGTTTATGATGTTGTACATTTTAATTTTGTCTACTTTTACTGGTTATGGTTTGTTGA
TGTTAGGTTGAGGTTCTTGGAAAAAGTATAGATTGATTAGTGCTGTTCGTGTGGCTTTTGCTAGAATAAGGTT
TGAAGCTACTTTTATGTGTTTAGTATTAGTATTGGGTGTAATATATAATGATTATGGTAAATTGGATATTACTTATT
TGATTGTTATTGCTCCATTAAATTATTTTGCTTGGTCTGTTTCTTTGCTTGCTGAAAGAAAACGTTCTCCATTTG
ATTATGGTGAGTCCGAGAGTGAATTAGTTAGCGGTTTAAATACTGAGTATAGTGGTTTATCATTTATAGTGATA
TTTGCTTTTGAATATGTAATGATGTTTATATCAAGATGAATTACTTCAATAGTTTTTTATGGAAGTTATATTGGTT
TAATTATTTTTCATTTGTTTTTATTTATTTGAGCACGTGGTACATTTCCGCGCGTTCGTTATGATTATTATGTGGC
AGTAGTATGAAAGTATGGTTTAGTTGTCTCTTTAATATATTTGTTAAGATGTTACGGATTGATTATAATTATAAG
GTAGTTTATGTAGATTAATTAAGATTGTGAGGCTGTTAACCTTAAGGTGAGTGTAAATTCCATAATCGTTTAAT
TTTAGATTATATATTCAGTTAATATTTTATTTAGAATATAAACTTTGGGTGTTTAAGGATTTGAATTTTCAAATTA
GCTGATTATTACGTTACTTATATGACTTGTTGTTATATTTAGCCGAGAGGGCTGCTAAGCAGGTTACTCCGATAT
AGTAAAATGTAAGAAAGACTTTTTTCCTCGGTATTTTTGTAATGTTTCATAGATATCTTAAGTATAAAGTGGAG
GATTCTTAATTCTTTGATGTTTAATTTATATAAACTCTGTGAGGTTATGTATATGTATTTTTTTTTTTTTTTTGGTG
TTTTATTTATAGTTTTAGTTGTTCGCTTTTATATGTTTTATTATTGGGGTTATAAGAATTTAGATTATAAGATTGGT
CGGGGTAAATGAGTTGATTCATTTGAGTGTGGTTTTATGACTCATGGGTTTAGGGAGAATTTTTTTAGTTTTT
CTTATTTAAATCTTTTGGTTTTTTTTGTTATATTTGACTTAGAGATATCTTTGTTATTAAATGTTCCTTTTGATGGT
GTTTGATATAATAGATTCTTTTGTTATATGGTTTTTATGGTGATGATTTTAATTATGTATATAATTGAAGTTTATTAT
GGTTTTGTTACTTGAACCAATTAGTATTGGTTTAGCTAAATAGGTTAGTTTGACTGTCTGTTTTCAAAACGGG
AGGTGGTTACGTATGATGTAGTCTTTAGTTGGATTTAGGTTTGGTTTAGTGGCGTAATATTTTAATTTAAAATAT
TTGTTTTACACACAAATGATGGGTAATTTACTCTGTCGCTATAAGTATCGGAAGTAATATATATTATCGCTGCTA
ACGATTGATTTGAAGGTTGTAAAATCTTCTACTTTCTTAATAAGTGGATTGACGTAAGAATTTATATGAATTGTA
CTTAAAGATTTTGTATAGTTTAATTTATTTAATGTATCGGTTACGTTGGTGAATAGAGGATCAGTTTAAGTACGA
AAATAATAAGGATTTGATATCTTGATTTGTCTCTTTGGATCATAAGCGTGTTGGTGTTGTTTATATTATTCTTGG
AGTGTGGGGTGGATTTATAGGTTTAGGTTTAAGTTTGTTGATACGTTTAAATTTTTGTGATCCTTATTATAAAC
TTATTCCTTGTGAGGTATATAATTATTTGATAACTAATCACGGTATAGCAATGATTTTTTTTTTTTTAATGCCTGT
TTTAATAGGTGGTTTTGGTAAATATCTTCTTCCGTTTTTTTTGAGTATGAGTGATTTAGCTTTGCCTCGTTTAAA
TTCTTTGAGTGTTTGAATGATGGTTCCTTCAATATTTTATATGGAATTGAGTTTGTATTATGGATCTGGTGTTGG
TTGGACCTTTTATCCACCTTTGTCTTCTTTAGCTACTTCTGGTGTTGGTGTGGATTACTTAATGTTCTCTTTACA
TCTTGCTGGTGTATCTAGTTTGATTGGTTCTATAAATTTTATTACTACTATAATGTTGCGTCTAAGGTCATGTTCT
TCAGTTATTAGATGATCTTATTTATTTACTTCGGTGTTGTTATTGTTATCGTTGCCGGTTCTTGCTGCAGGTATA
ACTATGTTGTTGTTTGATCGTAAATTTGGTACTGCTTTTTTTGAGCCAGCAGGTGGTGGTGATCCTGTGTTATT
TCAACATTTATTTTGGTTTTTTGGTCACCCAGAAGTATATGTTTTGATATTGCCTGGATTTGGTATAGTAAGTCA

TATATGTATGTCTTTAAGTAATAATAATTCTTCGTTTGGATATTATGGGTTAGTTTGTGCTATGGGTTCTATAGTG
TGTTTGGGGAGAGTTGTTTGGGCTCATCATATGTTTATGGTTGGTATGGATGTAAAGACTTCTGTTTTTTTTA
GTTCTGTAACAATGATTATAGGTATACCAACAGGTATTAAGGTGTTTTCTTGATTATATATGTTAGGGAGTAGT
GGGTTGCGTGCGGCTGATCCAATACTTTGGTGAATTGTTGGTTTTATATTTTTGTTTACAGTTGGTGGTGTTA
CTGGGATAGTTTTATCTGCTTCTGCTTTGGATAGATTATTTCACGATACTTGATTTGTGATAGCTCACTTTCATT
ATGTGCTGTCTTTGGGTTCTTATAGGAGTGTAATTATAATGTTTGTTTGGTGATGACCTTTTATAATTGGTTGTT
CTTTAAAAAAGTATTTGTTGCAAGGTCATTGATTATTGTCTATGGTTGGTTTTAATTTGTGTTTTTTTCCGATGC
ATTATTTAGGTGTTCATGGGTTGCCACGTCGTGTTAGATGTTATGATCCTGAGTTTTATTTGGTTAAAGTTTTTA
GGAGTATAGGTGGTGTGTTATCTGTAACTAGTTCGATGGTTTTTATGTTTTTATTATGAGAATCGTTGAGTGTT
TATAAATGTGTGTTAGGTTTATGAGGTTCTTGTGGGTGTGTATGTAATGTTCTAACTTGTCCTACATCTTATCATA
TTGATTATATAGCTGACGGTAAGAATTGATCAAAGTATTAGATTATAATAGTGTGTGTGTTTAATAGGTAAATTG
ATATGATTGTAGTAGTTATATAAGGATACATAGTTTAAGGTAAAATGATGCTTTTGTAAAGCATTGATAGATGAA
GTATTTTCTTGTTTTCATTTAAGATTAGAGTCTGCTGTAAATAGTTTTGTCTTTGGTTAAAGTACCTTTTGCATC
ATGATTCTTTGAGATGTTTAGATTATTTCTAGTTCCCGAATGGCTTATGATTTTCATTGATTCATAGCAAGTTTG
TGCATAGATAAGTTGTTGTGTAAACATTTGTAGAAATCATTGAGGTTGTGATAAATAATTCGTATAGTCTGATAA
CTGGTTTATAGCTTGATTATTTCAACGAAATAAAGATTGATTGTGTTTATCTTTACTGATAGAGATAGTTTATAT
GTTGTTGTTGAGTCTTGGATTAGAATTGTCCTAGGATAAGTTAGTTATTTACTTGACATTTTATTTGTATGTTTT
CTTATTGTTAATGGCTATATTCTTAATTATTTGTTTAATAATGAAGAAATGAGTAGGATTATGTTTTATTAGTCCG
TTATTTGTCTTTAATTCTGTTCAACTGTTTATCAAAAACATTGCTATTTGTTGGATTTAAATAGTATGGCCTGCCC
AATGTTGTAAATTAATGGTCGCAGTTTTACTGTGCTAAGGTAGCATAATATATCGCTTCTTAATTAGTGGCTTGT
GAATGGTTTAATGAAATAGAATATTTAAATGATGACTATATCTGAAATTGATTTAGTGGTGCGGAATCCATTGTT
ATAATATAAGACGGAAAGACCCCGAGATCTTGAATTAATTTTCTAATTTTGTTTGGGGTAAATGTATATATTTCA
TTTATTTTTAGATCCTATATATTGATGGATATAGGTTAAAGTTACCTCGGGGATAACTGAGTAAAGAGTTGTGA
GAGGTCTTATTGATCAACTTGTTACTACCTCGATGTTGGCTTAAAGAACCTTTATGGTGTAGCAGCTATATAAG
GTGGTCTGTTCGACCATTAAATCTTTATGAGTTGAGTTAAGACCGGTGTAAGCCAGGTCGGTTCTTATCTATA
AGTGAATCTAGTTAGTACGAAAGGAATATTAGAGTGTAAGATGTTGCATGTTTAATGTTAGAATACGTAATTAG
TATACTCTGCAAAAGTATAGATAGTAATGATGTTTTACTCGTATTTTTATACAATTTAACCCTGGTTGAAGTTGA
AATATATCTAATGATGCTATATACGTTTATTATATTATGCTAATCCTTGAGCATATAAATTTGAGGTGGTTGTAGTA
GTGAGATCTATCGTTATAAAGAAATTTAGTGATGGTTGTTAGATAGTAGAAGAGGCTAAAGCACAGTGCCAG
CAGCCGCGGTTATACTGTAATTCTTCTTATTTCTTTTTTGGTTAAAACTTTTATTGAAATTTAAATAAAATTGGTT
GGTTAAATAGGTTTTTTTTTGAGAATACTTCAATGATGTGTAAAGTTCCAGTATAAAGACAGGGATTAGATAC
CCCTTTATTAGGATAAAAAATTTTTGTCCACGGTTTCAAACTGAAAGGGTTTGGCAGCAAACTAAATTCTTCC
GGGGGAAGGTGCTTATTAAAGAGATGATCCGCTTATTAATTTACTATATCTAGTGTTAGTGTATATCCGTTTGTA
TATTCACGCTTAGAGGTCATAAGTGGGTAAATATTAAATAATTTAAGGCAGGTCTATATGCTGCTAATGGTATA
GGTTGTTGTGCGTTACAATAAAAAGTTTAAATACTGAAATGTATTTTTATTTTGGGACTCGGAAGTAGGAATG
TAAATAGTATGTCATTTCGAAGGTTGAAATAGTTTGGGTACACATCGCCCGACAATCTCGATGCGTAATTGAG
TTAAGTCGTAACATGGTAGTGCTTGAAGAATCAGGCGCTAGAATCTGTATAATGCGTTTAAAACATGAGCTTT
TAATTTATTATGACCTAGTTAAATACGTGTTATTTTTGTGTTGTTTTATTCCACTGTGGTGTATGATAGTGATGTG
CTATCAAATATATAATTCCCGCAAAATGAGAATAATGTTACCTAATGAAAGTCCATTTTTAGAGTTTATTTGAAC
ATTGATACCTACACTTATGGTTGTGATTTTATGTTTTTTTAAATTAAATTATTTAATTTATAATAGAGTTCAAGTTA
TGAAAGAACCAATAAAGATAGTTGGCCATCAGTGATATTGAACATACGAGTTGTTGGATGGTAGGGTTTATGA
TTCATTTATGACTGATTTTGTTAAAGGGGTTAATAAGCCGTTACGCTTAGAGCGTAATATTTCTTATATTTTATTA
ATTACATCTGATGACGTTATTCATTCTTTTTCTGTTCCTGATTTGGGTTTAAAGATGGATGCTATACCAGGTCGA
ATCAATAGTGTTATAGGTAGATTTGATCGTTTTGGTGTCTTCGTCGGTTATTGTACTGAATTATGTGGTGCTGG

TCATAGGTATATGCCTATAGTAGTTGAAGTTGTTGAGGGTAATCGGCTTGGATGATATTAATGTTTAGATAATGA
TTACTTCATTATTTTGTATAACTTATTTTATTTGTATGTATTGTTTTACGTTTAGAGGAAGGTCATTATTTCGTGTT
TTTTTAATAGTGGTAAGGTCCTTTAGTGTTGGAATGTTTATTTTTTATTGCAGTGGGTCTAGTTGATATTTTTTA
CTATTTGTTTTAATTTATTTTGGTGGTGTTTACATATTGTTTGTTTATATCAGAATGATGCTTCCTAAATCAGGTG
TATCAAGTATGAGGTTTAGGTATTTTTCTTTCTTGTTGGTATTTTTTTTTTTTTTTTTTTGTTTTTTTTTTTGCTAT
GAATTTTTAGGTACTAAAGTTATTGAGTATAGATTTTACTTGTGTAGAGATTTTGAGGCTGTTGTTTATAAATTT
TTTTGTTTGATATTATTATTTGGTATGTTTATAGTCAGTTTAATGATAAGAAGAGTTAAAGAGTATAGTCGGTAG
GATTATATATGTACATACGTATATTTAGCTATTTTAAGTTAGTACATTAATTAGTGTGTGTAGGTTGAAGTTTAAT
CGGCTTAGTATAAGAGTTTAATACTAGAAATTGTAAATTTCTAAATAGGTTATTCCTAGTCGAAATGCTAGTTAG
TTTTGTAAGATGCCAGATTTGATGGGTAGTGTTTAGGATTCTATTATGGGACTAAAAGAAGTTTCTCTTACAA
GCGTGTAAAGTGTTTTACATTGTTTTAATTTGAAGTTAATTTATCATGATTAATTTCATGTATACGCTTAGTGAG
TTAAACTTAGTTAAAATTTGTTTTTTGTAATTAAGTTATCTTAACATTTAATTAAGGGTTGATTTGGTTTTTACTA
AGTAAGCTAGTAATTAATGTATTAACTGATTAAACTAATAACATTTGTACAAGTGCCAGAGTAGTTATGGGTTG
GCTTTAAGCGTCAAATATAGACAGTTATGTCTCTTGTGTT
